# Induction of cell death by the novel proteasome inhibitor marizomib in glioblastoma *in vitro* and *in vivo*

**DOI:** 10.1038/srep18953

**Published:** 2016-01-25

**Authors:** Christa A. Manton, Blake Johnson, Melissa Singh, Cavan P. Bailey, Lisa Bouchier-Hayes, Joya Chandra

**Affiliations:** 1 Department of Pediatrics Research, University of Texas MD Anderson Cancer Center, Houston, TX 77030; 2 Graduate School of Biomedical Sciences, University of Texas Health Science Center, Houston, TX 77030; 3 Department of Pediatrics, Baylor College of Medicine, Houston, TX 77030.

## Abstract

New therapies for glioblastoma (GBM) are needed, as five-year survival is <10%. The proteasome inhibitor marizomib (MRZ) has inhibitory and death-inducing properties unique from previous inhibitors such as bortezomib (BTZ), and has not been well examined in GBM. We evaluated the mechanism of death and *in vivo* properties of MRZ in GBM. The activation kinetics of initiator caspases 2, 8, and 9 were assessed using chemical and knockdown strategies to determine their contribution to cell death. Blood brain barrier permeance and proteasome inhibition by MRZ and BTZ were examined in an orthotopic GBM model. Blockade of caspase 9, relative to other caspases, was most protective against both MRZ and BTZ. Only MRZ increased the proteasome substrate p27 in orthotopic brain tumors after a single injection, while both MRZ and BTZ increased p21 levels after multiple treatments. Cleavage of caspase substrate lamin A was increased in orthotopic brain tumors from mice treated with MRZ or BTZ and the histone deacetylase inhibitor vorinostat. Our data indicate that MRZ induces caspase 9-dependent death in GBM, suggesting drug efficacy biomarkers and possible resistance mechanisms. MRZ reaches orthotopic brain tumors where it inhibits proteasome function and increases death in combination with vorinostat.

Glioblastoma multiforme (GBM) is an aggressive form of brain cancer with a median survival of 14 months^1^,^2^. New therapeutic strategies are needed for GBM, and one approach involves targeting the proteasome, the complex responsible for the bulk of protein degradation in cells. Proteasome inhibition leads to toxic accumulation of misfolded and abnormal proteins in cells and can also stabilize specific tumor inhibitory factors such as cell cycle regulatory proteins and pro-apoptotic factors^3^,^4^,^5^. Proteasome inhibitors have been shown to activate cell death in different types of cancer, including multiple myeloma and leukemia, in a manner dependent on activation of caspases and apoptotic cell death^6^,^7^,^8^.

The proteasome inhibitor bortezomib (BTZ) is FDA-approved for multiple myeloma and mantle cell lymphoma^9^ and has been evaluated clinically in GBM. Other proteasome inhibitors have also been developed, including marizomib (MRZ, formerly NPI-0052)^10^,^11^. As an irreversible inhibitor, MRZ is unique from BTZ, which is a slowly reversible inhibitor^12^,^13^,^14^. While both MRZ and BTZ target the chymotryptic-like activity of the β5 proteasome subunit, they target the other catalytic activities to different extents, with MRZ inhibiting the trypsin-like activity of the β2 subunit more strongly than BTZ^15^. Also, MRZ is more dependent on caspase 8 than BTZ in myeloma and leukemia^8^,^16^ and MRZ induces death in BTZ-resistant myeloma cells^15^, demonstrating that these inhibitors can trigger different death pathways. A phase I study of MRZ in relapsed multiple myeloma is currently ongoing, and there are plans to initiate a phase I trial in GBM^17^.

*In vitro* studies have mainly utilized MTT assays to demonstrate that MRZ and BTZ cause death in GBM cell lines^18^,^19^. Also, BTZ induces cleavage of poly(ADP ribose) polymerase^20^, suggesting a caspase-dependent mechanism of death. However, the mechanism of death induced by MRZ, and its dependence on specific initiator caspases, has not been reported in GBM. This information could aid in design of combination strategies that potentiate apoptosis, identify biomarkers of drug efficacy, and help anticipate drug resistance^21^.

Another important question concerning MRZ utility for GBM treatment involves drug delivery to brain tumors. The blood brain barrier (BBB) may prevent drug delivery to brain tumors, while BBB disruption by brain tumors may facilitate drug delivery^22^. Though a previous study indicated that MRZ did not decrease proteasome activity in the brain, this was in mice without brain tumors, and therefore with intact BBB^10^,^23^. It is important to use orthotopic tumor models to answer these questions. This issue may explain the mixed results with BTZ *in vivo*. Despite strong *in vitro* efficacy^24^,^25^, the combination of BTZ and the histone deacetylase inhibitor (HDACi) vorinostat failed to prevent progression in GBM patients in a phase II clinical trial^26^. This trial did not include molecular markers to indicate whether BTZ successfully inhibited proteasomes in brain tumors at the dose and treatment schedule used. This illustrates why proteasome inhibitors with unique properties, such as MRZ, should be carefully studied to define their *in vivo* dynamics.

Past *in vitro* and clinical experience with proteasome inhibitors has demonstrated important needs in two avenues of research: 1) determining events necessary for proteasome inhibitor efficacy that can serve as biomarkers and 2) testing next-generation proteasome inhibitors such as MRZ that may have unique delivery, inhibitory, and death-inducing properties leading to enhanced clinical efficacy. Therefore, the goals of this study were to establish the pathway of cell death induced by MRZ in GBM and to evaluate its ability to affect changes in proteasome substrates and death induction in combination with vorinostat in an orthotopic GBM model.

## Results

### Proteasome inhibition by pulse treatment with MRZ *in vitro* and *in vivo*

**Figure 1:**
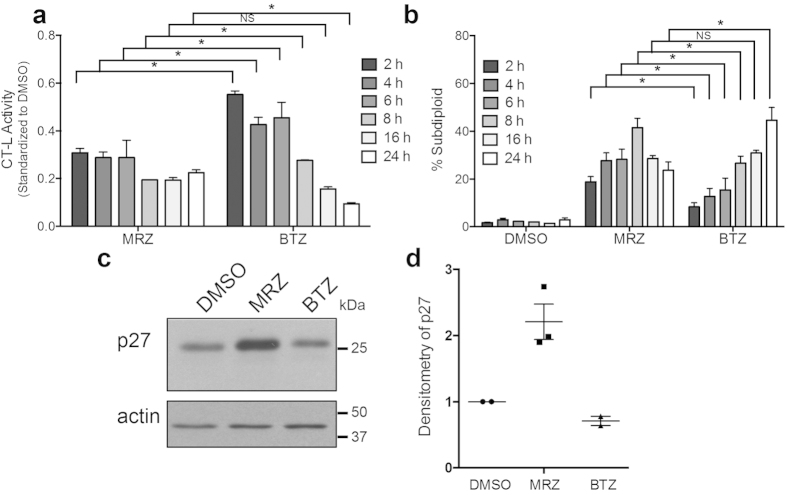
Proteasome inhibition and death induction by pulse treatment and single injection of MRZ *in vitro* and *in vivo*. (**a,b**) LN18 GBM cells were treated with 100 nM MRZ or BTZ for the times indicated in the figure. After that time, the drug was removed, wells were washed with PBS, and fresh media was added until a total time of 24 h had elapsed before measurement of chymotrypsin-like proteasome activity (**a**) or 48 h had passed before assessment of DNA fragmentation (**b**). *p < 0.05, NS = not significant. (**c**) Levels of p27 in lysates from the tumor-bearing portion of the brains of mice with orthotopic brain tumors that developed for 7 days before single IP injection with 1.0 mg/kg BTZ or 0.15 mg/kg MRZ 24 h before sacrifice. (**d**) Densitometry quantification of individual mice from (**c**) (DMSO and BTZ, N = 2 mice; MRZ, N = 3 mice).

As an irreversible inhibitor of the proteasome, MRZ should be able to induce sustained proteasome inhibition after brief exposure. We examined proteasome inhibition and DNA fragmentation in LN18 GBM cells treated with short pulses of MRZ or the reversible inhibitor BTZ. Cells were treated with drug for the times indicated in the figure; after that time, wells were washed with PBS and fresh media was added until a total of 24 h had passed for measurement of proteasome activity (Fig. 1a), or 48 h had elapsed for assessment of DNA fragmentation (Fig. 1b). MRZ treatments as short as 2 h caused proteasome inhibition that was sustained out to 24 h. However, longer exposure to BTZ was necessary to achieve the same effects. Shorter MRZ treatments also induced DNA fragmentation, while BTZ required longer exposures.

To study this effect *in vivo* and to establish the inhibitory capacity of MRZ in an intracranial brain tumor model, mice with orthotopic U87 cell brain tumors that had developed for 1 week were treated with a single intraperitoneal injection of MRZ or BTZ. Doses of MRZ and BTZ (0.15 mg/kg and 1.0 mg/kg, respectively) were chosen based on established maximum tolerated doses^10^,^27^. Twenty-four hours after treatment, we observed increased levels of the proteasome substrate p27 in lysates from the tumor-bearing portion of the brain of MRZ-treated mice (Fig. 1c,d).

### Sustained proteasome inhibition yields increased death in GBM *in vitro* and *in vivo*

**Figure 2:**
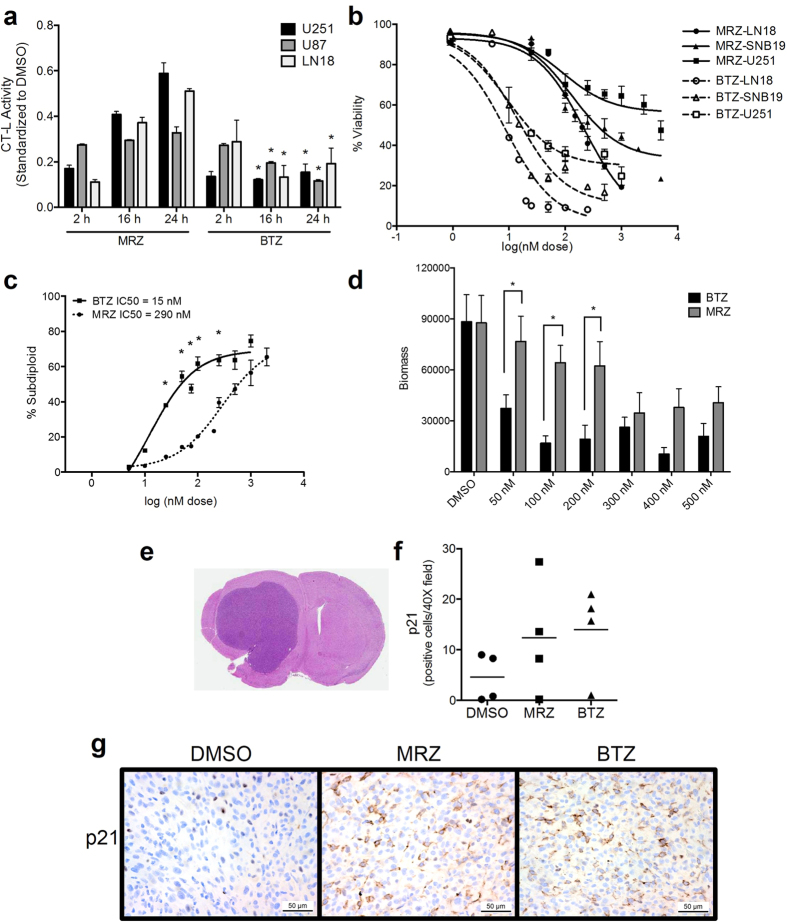
Proteasome inhibition after continuous treatment *in vitro* and repeated injection *in vivo.* (**a**) Chymotrypsin-like proteasome activity in LN18, U251, and U87 cells treated with 75 nM BTZ or MRZ (*p < 0.05 compared to MRZ). (**b**) Viability measured by trypan blue permeability after 48 h treatment with BTZ or MRZ. (**c**) Percentage of LN18 cells with DNA fragmentation after 48 h treatment with BTZ or MRZ. (**d**) Biomass of LN18 colonies after treatment with proteasome inhibitors for 3 days (*p < 0.05). (**e**) Representative H&E staining from a DMSO-treated mouse with an orthotopic brain tumor that developed for 14 days. (**f,g**) Quantification of the average number of p21-positive cells per 40 × field (**f**) and representative images of p21 staining (G, 40×) in brain tumors from mice with U87 tumors that had developed for 14 days, followed by IP injection with either BTZ (1.0 mg/kg), MRZ (0.15 mg/kg), or vehicle (DMSO) twice per week for 2 weeks. Brains were harvested 24 h following the final treatment. All error bars represent the standard error of the mean for N = 3 independent experiments. NS= not specific.

In addition to our examination of single pulse treatments (Fig. 1), we also examined the action of MRZ and BTZ in a panel of GBM cell lines after continuous treatment. Both agents caused strong initial proteasome inhibition in GBM cells, though MRZ-treated cells recovered proteasome activity between 16 and 24 h, whereas BTZ caused more sustained inhibition (Fig. 2a). Treatment with the vehicle (DMSO) did not significantly impact proteasome activity (Supp. Fig. 1). Blocking drug efflux with verapamil, an inhibitor of P-glycoprotein, did not enhance the activity of either drug, suggesting that efflux does not account for the recovery after MRZ treatment (Supp. Fig. 2). Viability measurement using trypan blue demonstrated that GBM cell lines were sensitive to both MRZ and BTZ at nanomolar doses (Fig. 2b). LN18 cells treated with MRZ and BTZ also had increased DNA fragmentation (Fig. 2c) and decreased colony growth (Fig. 2d).

The increased death caused by BTZ in Fig. 2b–d suggests a link between more sustained proteasome inhibition (Fig. 2a) and increased death. To examine this effect *in vivo*, mice with orthotopic U87 brain tumors that had developed for 2 weeks were treated for a more continuous period: twice weekly for 2 weeks (representative H&E stained control brain with tumor, Fig. 2e). There was an increase in the number of p21-positive cells in tumors from MRZ- or BTZ-treated mice (Fig. 2f,g). Therefore, both MRZ and BTZ affect proteasome substrates *in vivo* after multiple treatments.

**Figure 3:**
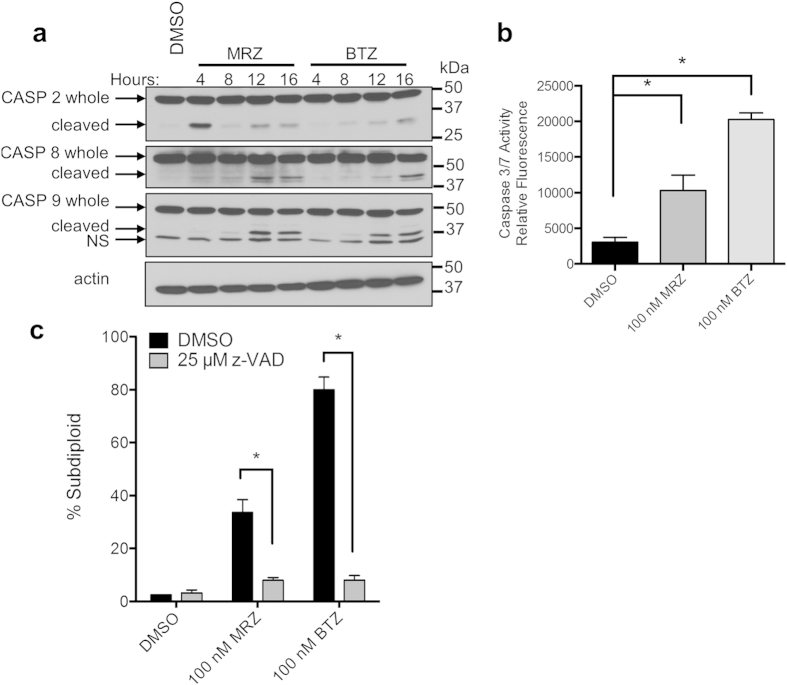
Caspase dependence of death induced by MRZ and BTZ in GBM cells. (**a**) Cleavage of caspases 2, 8, and 9 in LN18 cells treated with 100 nM BTZ or MRZ. (**b**) Caspase 3/7 activity in LN18 cells treated with BTZ or MRZ for 16 h. (**c**) DNA fragmentation in LN18 cells pre-treated for 30 min with a pan-caspase inhibitor (z-VAD-fmk), followed by 48 h treatment with BTZ or MRZ. *p < 0.05. All error bars represent the standard error of the mean for N = 3 independent experiments.

Proteasome inhibitors induce caspase-dependent death in leukemia^8^. To assess whether this was also true in GBM, we examined initiator caspases 2, 8, and 9 after treatment with MRZ or BTZ. All 3 caspases were cleaved, a step in the activation process, after treatment with BTZ and MRZ (Fig. 3a). Caspase 2 was cleaved after 4 h, particularly with MRZ, whereas caspases 8 and 9 were cleaved later (8–12 h). Executioner caspase 3/7 activity was detected 16 h after treatment (Fig. 3b). Nearly all DNA fragmentation induced by BTZ and MRZ was blocked by pre-treatment with the pan-caspase inhibitor z-VAD-fmk, and viability was also increased (Supp. Fig. 3). This indicates that caspases play a crucial role in death induction by proteasome inhibitors in GBM cells (Fig. 3c).

### Early cleavage of caspase 2 by proteasome inhibitors is not essential for death

**Figure 4:**
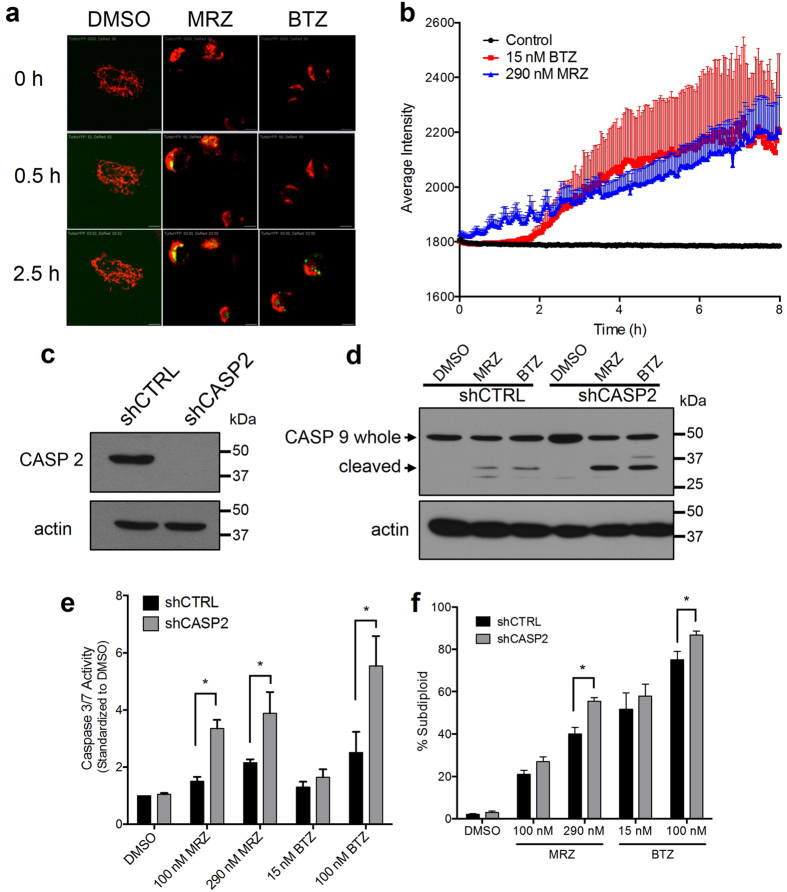
Caspase 2 is activated early and suppresses death induced by MRZ and BTZ. (**a**) Images of BiFC-Venus caspase 2 fluorescence (*green*) following treatment with 290 nM MRZ or 15 nM BTZ (IC50 doses) (*red* = dsRed mito). (**b**) Quantification of average Venus BiFC intensity in LN18 cells from (**a**) (DMSO [N = 8 cells], BTZ [N = 14 cells], MRZ [N = 13 cells]). (**c**) Caspase 2 protein in LN18 cells stably expressing shCTRL and shCASP2. (**d**) Activation of caspase 2 in shCTRL and shCASP2 LN18 cells after 16 h of treatment with 100 nM MRZ or BTZ. (**e**) Caspase 3/7 activity in shCTRL and shCASP2 LN18 cells after 16 h of treatment with MRZ or BTZ. (**f**) DNA fragmentation in shCTRL and shCASP2 LN18 cells treated for 48 h with MRZ or BTZ. *p < 0.05. All error bars represent the standard error of the mean for N = 3 independent experiments.

After observing early cleavage of caspase 2, we used a more direct method to confirm that this caspase 2 cleavage represented caspase 2 activation. We measured recruitment of caspase 2 to activation platforms, the initiating step in its activation, using a Venus bimolecular fluorescence (BiFC) model that measures induced proximity of caspase 2 (Fig. 4a). For this experiment, we used doses of MRZ and BTZ (290 nM and 15 nM, respectively) that were equipotent, meaning that they resulted in DNA fragmentation in 50% of the cells after 48 h (Fig. 2c). Both MRZ and BTZ induced caspase 2 BiFC, with MRZ inducing caspase 2 activation slightly earlier than BTZ (Fig. 4b).

To examine the importance of caspase 2 in proteasome inhibitor-induced death, we generated LN18 cells stably expressing caspase 2 shRNA (Fig. 4c). Active caspase 2 has been shown to induce mitochondrial membrane permeability and activation of caspase 9^28^,^29^. However, shCASP2 cells treated with MRZ or BTZ had increased cleavage of caspase 9 (Fig. 4d), increased caspase 3/7 activity (Fig. 4e), and slightly increased overall DNA fragmentation (Fig. 4f). In addition to the stable knockdown of caspase 2, we also observed that transient knockdown with siRNA against caspase 2 did not impact induction of death following proteasome inhibition (Supp. Fig. 4). Therefore, caspase 2 does not appear to be essential for death induction following proteasome inhibition in GBM.

### Caspase 9 functions upstream of caspase 8 to induce death after proteasome inhibition

**Figure 5:**
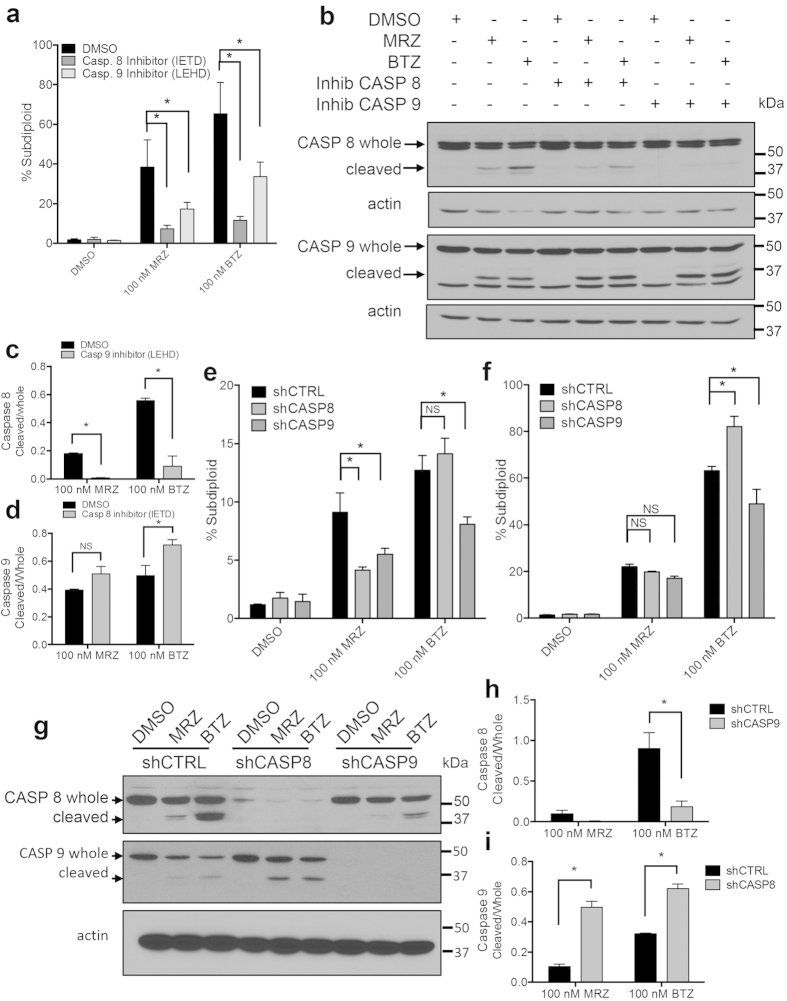
Chemical inhibitors and shRNA against caspases 8 and 9 reveal that inhibition of caspase 9 prevents activation of caspase 8 in cells treated with proteasome inhibitors. (**a**) DNA fragmentation in LN18 cells pre-treated 30 min with caspase 8 inhibitor (25 μM z-IETD-fmk) or caspase 9 inhibitor (25 μM z-LEHD-fmk), followed by 48 h of treatment with proteasome inhibitors. (**b**) Western blot of caspases 8 and 9 in LN18 cells pre-treated with specific inhibitors of caspase 8 or 9, followed by 16 h treatment with 100 nM MRZ or BTZ. (**c,d**) Western blot densitometry from (**b**) showing the ratio of cleaved to whole caspase 8 in cells pre-treated with the caspase 9 inhibitor (**c**), or the ratio of cleaved to whole caspase 9 in cells pre-treated with the caspase 8 inhibitor (**d**). (**e,f**) DNA fragmentation in LN18 cells stably expressing shCTRL, shCASP 8, or shCASP9 treated for 24 h (**e**) or 48 h (**f**) with proteasome inhibitors. (**g**) Western blot of caspase 8 and 9 cleavage in shCTRL, shCASP8, and shCASP9 LN18 cells treated 16 h with 100 nM proteasome inhibitors. (**h,i**) Densitometry from (**g**) showing the ratio of cleaved to whole caspase 8 in cells with or without caspase 9 (**h**), or the ratio of cleaved to whole caspase 9 in cells with or without caspase 8 (**i**). *p < 0.05. All error bars represent the standard error of the mean for N = 3 independent experiments.

We next examined the initiator caspases 8 and 9, which were activated 8–12 h following proteasome inhibition (Fig. 3a). Pre-treatment with specific inhibitors of either caspase 8 (z-IETD-fmk) or caspase 9 (z-LEHD-fmk) significantly protected cells from both MRZ- and BTZ-induced DNA fragmentation (Fig. 5a). To identify the initiator of the caspase cascade, we examined whether chemical inhibition of either caspase 8 or 9 blocked activation of the other caspase. These inhibitors act as caspase substrates; after initial cleavage of the targeted caspase, the inhibitors bind the active caspase to prevent cleavage of downstream substrates. Therefore, initial cleavage of the targeted caspase will still be visible on Western blot, but binding of the inhibitor to the active caspase prevents further cleavage of downstream targets. Inhibition of caspase 9 prevented caspase 8 cleavage, while inhibition of caspase 8 did not diminish caspase 9 cleavage (Fig. 5b–d).

Caspases have overlapping cleavage site specificities, so chemical inhibitors are not specific for any one caspase^30^. Therefore, we confirmed our results in cells stably expressing shRNA for caspase 8 or 9. Twenty-four hours after proteasome inhibitor treatment, shCASP8 cells showed decreased sensitivity to MRZ, but not BTZ (Fig. 5e). Notably, shCASP9 cells were more resistant to both MRZ and BTZ, indicating that caspase 9 is important for initial death induction by both inhibitors. These effects were blunted after 48 h of treatment, indicating that late compensatory mechanisms may obscure early death events (Fig. 5f). However, the data at 24 h confirms that caspase 9 is important for initial death induction by both MRZ and BTZ.

Caspase activation was also examined in shCASP8 and shCASP9 cells treated with MRZ and BTZ (Fig. 5g). Caspase 8 activation was blocked in shCASP9 cells (Fig. 5h), while caspase 9 activation was not diminished in shCASP8 cells (Fig. 5i), confirming the result with the chemical inhibitors. Together, data from both chemical inhibitors and shRNA indicates that caspase 9 is at the top of the apoptotic cascade induced by both MRZ and BTZ in GBM cells.

### Caspase 9 activation and death are blocked by reducing agents

**Figure 6:**
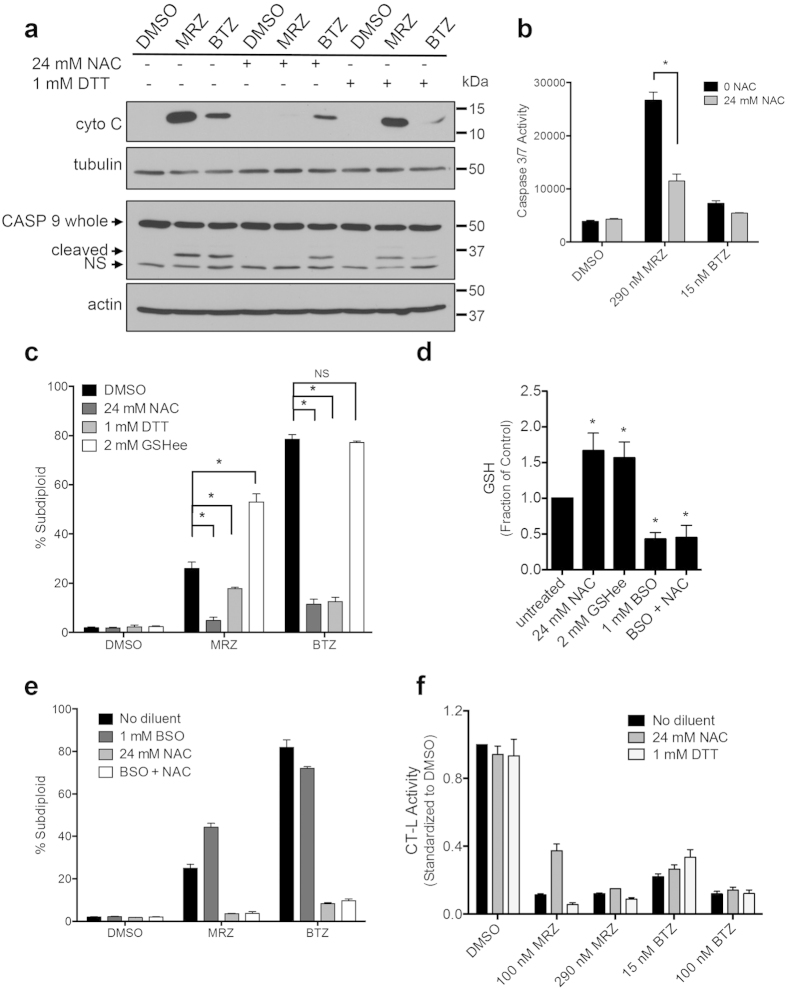
Reducing agents protect against proteasome inhibitor induced death independently of glutathione modulation or inactivation of proteasome inhibitors. (**a**) Cytochrome C in cytoplasmic fractions and caspase 9 cleavage in whole cell lysates in LN18 cells were pre-treated 30 min with 24 mM NAC or 1 mM DTT, followed by treatment with 100 nM proteasome inhibitors for 8 h (cyto C) or 16 h (caspase 9). (**b**) Caspase 3/7 activity in LN18 cells pre-treated with 24 mM NAC, followed by 16 h treatment with proteasome inhibitors (*p < 0.05). (**c**) DNA fragmentation in LN18 cells pre-treated for 30 min with NAC, DTT, or GSHee, followed by 48 h treatment with 100 nM proteasome inhibitors (*p < 0.05). (**d**) Levels of glutathione in LN8 cells treated for 6 h with NAC, GSHee, BSO, or BSO + NAC (*p < 0.05 compared to untreated cells). (**e**) DNA fragmentation in LN18 cells treated first with BSO (30 min), followed by treatment with NAC (30 min) and 48 h treatment with 100 nM proteasome inhibitors. (**f**) CT-L proteasome activity in cells pre-treated 30 min with NAC or DTT, followed by 4 h treatment with proteasome inhibitors. All error bars represent the standard error of the mean for N = 3 independent experiments.

In other cancer types, production of reactive oxygen species (ROS) is an integral part of proteasome inhibitor-induced death^8^,^31^,^32^,^33^. To study effects of ROS, we pre-treated cells with N-acetylcysteine (NAC), an antioxidant that increases cellular glutathione (GSH), or dithiothreitol (DTT), a general reducing agent. Pre-treatment with NAC or DTT diminished mitochondrial release of cytochrome C, cleavage of caspase 9 (Fig. 6a), caspase 3/7 activity (Fig. 6b), and DNA fragmentation (Fig. 6c) in LN18 cells treated with BTZ and MRZ. Reductions in caspase activation were more pronounced in cells treated with reducing agents and MRZ compared to BTZ.

Since the antioxidant mechanism of NAC is widely considered to be through increased GSH, we also pre-treated cells with cell-soluble glutathione ethyl ester (GSHee). Though GSHee and NAC similarly raised GSH levels (Fig. 6d), GSHee did not prevent proteasome inhibitor-induced DNA fragmentation (Fig. 6c). Additionally, inhibition of GSH synthesis by buthionine sulphoximine (BSO) depleted GSH (Fig. 6d) but did not prevent NAC from protecting cells from proteasome inhibitors (Fig. 6e). Importantly, NAC and DTT did not attenuate the ability of BTZ and MRZ to reduce proteasome activity (Fig. 6f). These results indicate that the reducing agents NAC and DTT may protect from proteasome inhibitor-induced death by preventing upstream apoptotic events through GSH-independent mechanisms, without affecting the proteasome inhibitory activity of BTZ and MRZ.

### HDACi synergize with proteasome inhibitors *in vitro* and *in vivo*

**Figure 7:**
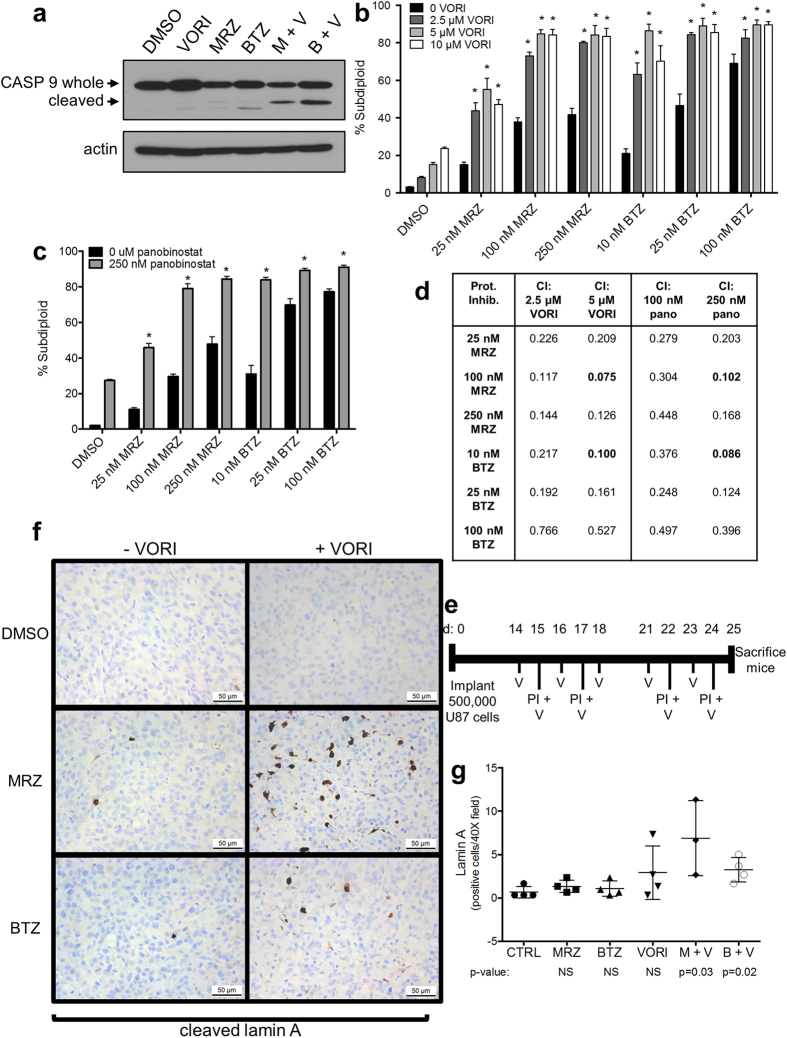
Combination proteasome inhibition and HDAC inhibition induces increased caspase-9 activation and DNA fragmentation *in vitro* and cleavage of the caspase substrate, lamin A, *in vivo*. (**a**) Caspase 9 in LN18 cells treated 16 h with 100 nM MRZ, 10 nM BTZ, 5 μM vorinostat, or combinations. (**b,c**) DNA fragmentation in LN18 cells treated with combinations of proteasome inhibitors and vorinostat (**b**) or panobinostat (**c**) for 48 h (*p < 0.05 compared to either single agent alone). (**d**) Combination index values for combinations of proteasome inhibitors and HDACi. Values <1 indicate synergy. Most highly synergistic doses are in bold font. (**e**) Treatment scheme for mice treated with combinations of proteasome inhibitors and vorinostat. Tumors developed for 14 days, followed by treatment for 2 weeks with IP injections of BTZ (1.0 mg/kg twice weekly), MRZ (0.15 mg/kg twice weekly), or VORI (50 mg/kg 5 times per week) before mice were sacrificed 24 h following the last treatment. (**f**) Representative images of IHC staining for cleaved lamin A in tumors of mice treated for 2 weeks with combinations of BTZ, MRZ, and VORI (40 × magnification). (**g**) The average number of cleaved lamin A-positive cells per field for individual mice treated as in (**e,f**). p-values indicate significant differences compared to the control group, 40× magnification. All error bars represent the standard error of the mean for N = 3 independent experiments.

Development of combination treatment regimens is essential for a viable clinical strategy. Previous studies in other cancer types have indicated that combining proteasome inhibitors with HDACi can be a potent therapeutic strategy^8^,^34^. BTZ plus the HDACi vorinostat induced a strong reduction in mitochondrial membrane potential in GBM cells^25^, and we found that this combination amplifies caspase 9 cleavage (Fig. 7a). We analyzed DNA fragmentation in cells treated with either BTZ or MRZ and two different HDACi: the FDA-approved inhibitor vorinostat (Fig. 7b) and a newer HDACi, panobinostat (Fig. 7c). Using CalcuSyn software to analyze our DNA fragmentation results, we identified multiple synergistic combinations for BTZ and MRZ and both HDACi (Fig. 7d).

We next examined this combination *in vivo.* Mice with intracranial tumors were treated by the schedule outlined in Fig. 7E, and tumors were examined for cleaved lamin A, a caspase substrate (Fig. 7f). Mice treated with MRZ or BTZ plus vorinostat had significantly increased cleaved lamin A-positive cells compared to control mice (Fig. 7g).

## Discussion

This is the first study to evaluate functional effects of MRZ in an orthotopic GBM model. We demonstrated enhanced proteasome inhibition by MRZ after pulse treatments and single injection *in vitro* and *in vivo*, which could be beneficial for integrating this agent into a chemotherapy regimen. Though MRZ was less potent than BTZ after continuous treatment *in vitro*, our *in vivo* data suggests a different dynamic in an orthotopic model, perhaps due to better BBB penetration of MRZ. Future studies using radiolabeled MRZ in orthotopic models would be required to adequately answer this question.

This study also found that MRZ and BTZ induce caspase 9 initiated apoptosis in GBM cells. Events specifically related to this pathway may be useful in future clinical trials to aid interpretation of efficacy results and anticipate and overcome drug resistance. These results also indicate that MRZ acts differently in GBM cells compared to myeloma and leukemia, where it was shown to be dependent on caspase 8^8^,^16^.

Though we were able to show caspase 2 activation by 2 methods, we found that knockdown of caspase 2 did not impact death induction by proteasome inhibitors. Previous reports have described a role of caspase 2 as a tumor suppressor, and have found that cells deficient in caspase 2 show increased proliferation and defective cell cycle checkpoint regulation after DNA damage^35^. Therefore, caspase 2 activation may have interesting functions that warrant future investigation.

In addition to caspase 9 inhibition, treatment with NAC or DTT also blocked caspase activation and cell death in a manner independent of GSH. NAC and DTT may impact thiol levels to create a reduced cellular environment. Thiol modulation can impact cellular stress responses, as DTT and NAC have been shown to prevent heat shock death in endothelial cells, whereas GSH was not protective^36^. Given their broad effects on the cellular environment, NAC and DTT may affect many proteins in stress response and cell death pathways. Vitamin C and other antioxidants have been shown to prevent BTZ efficacy in myeloma^37^, and thiol-rich agents should also be evaluated for contraindications. Our study suggests that GSH, but not NAC, may be an appropriate treatment to alleviate side effects such as BTZ-associated peripheral neuropathy^38^.

Combination strategies are key to clinical efficacy of these agents. MRZ and BTZ synergized with HDACi *in vitro* and induced cleavage of lamin A *in vivo*. There was a trend toward increased lamin A cleavage in mice treated with vorinostat plus MRZ versus the combination with BTZ, indicating once again that MRZ may be exerting a stronger effect than BTZ *in vivo*.

This study highlights the idea that new proteasome inhibitors and HDACi have improved targeting and clinical utility. Besides MRZ, this study also examined panobinostat, a novel HDACi that has shown promise in combination with BTZ in myeloma^39^,^40^. In the current study, panobinostat synergized with both MRZ and BTZ at lower doses than vorinostat *in vitro*, indicating that this combination warrants future examination. Agents such as MRZ and panobinostat have promising preclinical properties, and they warrant careful examination to delineate their clinical potential. Future experiments in relevant models will also be able to assess the ability of MRZ to impact tumor size and survival as part of various therapeutic combinations.

## Materials and Methods

### Cell lines and reagents

All GBM cell lines (LN18, SNB19, U87, and U251) were obtained from ATCC. The cells used possess varied genetic characteristics: U87 cells are wild-type for p53, while SNB19, U251, and LN18 express mutant p53; LN18 cells express wild-type PTEN, while U87, SNB19, and U251 cells have altered PTEN status. Cells were authenticated by the Characterized Cell Line Core Facility at MD Anderson Cancer Center using the short tandem repeat method. Cells were maintained in an incubator at 37 °C with 5% CO_2_ in DMEM/F12 media with 10% FBS, 1% penicillin and streptomycin, and 1% L-glutamine. BTZ was obtained from LC Labs (Woburn, MA) and MRZ was provided by Nereus Pharmaceuticals (San Diego, CA).

### Proteasome activity assay

Cells were washed 1× with PBS and resuspended in 20S proteasome lysis buffer (20 mM Tris, pH 7.5, 0.1 mM ethylenediaminetetraacetic acid, 20% glycerol, and 0.05% NP-40 supplemented each time with fresh 1 mM β-mercaptoethanol and 1 mM adenosine triphosphate). Cells were lysed by freezing and thawing 3× on dry ice. Samples were then spun for 1 min at 12,000 rpm. Samples were aliquoted to duplicate wells (100 μL/well) of a black 96-well plate. Next, 98 μL substrate buffer (50 mM HEPES, pH 7.5, and 50 mM EGTA, pH 7–8) were added to each well along with 2 μL suc-LLVY-amc fluorogenic substrate for chymotrypsin-like activity (AG Scientific, San Diego, CA, USA). After 1 h incubation with fluorogenic substrates, fluorescence was read on a Gemini EM Microplate Reader (Molecular Devices, Sunnyvale, CA, USA) at an excitation of 380 nM and an emission of 460 nM. For experiments that indicate they were standardized to DMSO, all samples were divided by the fluorescence value for control (DMSO treated) cells.

### Cell viability and DNA fragmentation

Viability was measured by trypan blue exclusion assay using a Vi-CELL® (Beckman Coulter, Inc., Pasadena, CA). For analysis of DNA fragmentation, cells were fixed in 70% ethanol for at least 24 h. Cells were then incubated with 50 μg/mL propidium iodide and 100 μg/mL ribonuclease A in PBS. Cell cycle was analyzed by flow cytometry (FACSCalibur™, BD Biosciences, San Jose, CA), and the subdiploid population was gated.

### Colony growth assay

Colonies were grown in soft agarose as described previously^41^. A base agarose layer was formed by mixing 10 mL sterile water containing 4% low-melt agarose with 85 mL DMEM/F12 media containing 10% FBS and 15 mL additional FBS. The base agarose (0.5 mL) was added to each well in a 24-well plate. Then, a top agarose layer was formed by mixing 10 mL sterile water containing 3% low-melt agarose with 42.5 mL DMEM/F12 media containing 10% FBS and 7.5 mL additional FBS. LN18 cells were added to the top agarose (1,300 cells/mL), and 0.5 mL top agarose containing cells was added to each well of the 24-well plate on top of the base layer of agarose. After the top matrix solidified, 300 μL warm media was added to the top of each well. Cells were grown in these plates for 5 days. After 5 days, proteasome inhibitors were added to the top media of each well in doses normalized to the volume of the agar plus top media, and cells were incubated with the drug for 3 additional days. Wells were analyzed using a GelCount^™^ machine (Oxford Optronix, Oxfordshire, England). Biomass was calculated as the number of colonies multiplied by the average volume.

### Western blotting

Cells were lysed for 1 h at 4 °C in Triton X-100 lysis buffer (PBS containing 1% Triton X-100, 25 mM Tris, pH 7.5, and 157 mM NaCl) supplemented with a cOmplete Mini protease inhibitor cocktail tablet (Roche, Basel, Switzerland) and 1 mM glycerol phosphate, 1 mM NaF, and 1 mM NaOrthoV. Debris was pelleted by spinning samples for 20 min at 12,000 rpm at 4 °C, and protein concentrations were determined by Bradford Assay (Bio-Rad, Hercules, CA, USA).

Proteins were separated by sodium dodecyl sulfate polyacrylamide gel electrophoresis and transferred onto polyvinylidene fluoride membranes. After blocking for 1 h at room temperature in 5% milk or bovine serum albumin in TBS-T, membranes were incubated with 1:1,000 dilutions of the following primary antibodies: actin (Sigma, St. Louis, MO, USA), caspase 8, caspase 9, tubulin (Cell Signaling, Beverly, MA, USA), caspase 2 (EMD Millipore, Billerica, MA, USA), cytochrome C, and p27 Kip1 (BD, San Jose, CA, USA). Membranes were then washed 3× with TBS-T before being incubated with appropriate horseradish peroxidase conjugated secondary antibodies (mouse and rabbit: GE Healthcare, Buckinghamshire, England; rat: Cell Signaling, Beverly, MA, USA). Chemiluminescent visualization of bands was performed using a Kodak film developer (Rochester, NY, USA).

### Caspase 3/7 activity assay

Cells in PBS were frozen and thawed on dry ice, then incubated for 3 h with 150 μL DEVD buffer, pH 7.25 (100 mM HEPES, 10% sucrose, 5 mM DTT, 0.0001% IGEPAL, 0.1% CHAPS) containing 50 μM Ac-DEVD-amc fluorogenic substrate (Enzo Life Sciences, Farmingdale, NY). Fluorescence was read on a Gemini EM Microplate Reader (Molecular Devices, Sunnyvale, CA) at an excitation of 355 nM and an emission of 460 nM.

### Bimolecular fluorescence complementation (BiFC) measurement of caspase 2 activation

Measurement of induced proximity of caspase 2 using the caspase-2 BiFC was performed as previously described^42^. LN18 cells were plated in a 4-chamber dish containing coverslips (Nunc, Roskilde, Denmark) 24 h prior to transfection. Cells were transfected with caspase 2-CARD-VC (100 ng/well), caspase 2-CARD-VN (100 ng/well), and dsRed mito (20 ng/well) using lipofectamine 2000 (Life Technologies, Carlsbad, CA, USA). The following day, cells were treated with 290 nM MRZ or 15 nM BTZ. Cells were imaged using a spinning disk confocal microscope (Zeiss, Jena, Germany) equipped with a CSU-X1A 5000 spinning disk unit (Yokogowa Electric Corporation, Japan), multi-laser module with wavelengths of 458 nM, 488 nM, and 514 nM, and an Axio Observer Z1 motorized inverted microscope equipped with a precision motorized XY stage (Carl Zeiss MicroImaging, Thornwood, NY, USA). Temperature was maintained at 37 °C and 5% CO_2_ using an environmental control chamber. Zen 2012 software (Zeiss) was used to acquire images using a Zeiss Plan-Neofluar 40 × 1.3 NA objective on an Orca R2 CCD camera and to analyze average Venus intensity.

### Specific chemical and shRNA inhibition of caspases 2, 8, and 9

Cells were pre-treated with specific inhibitors of caspase 8 (25 μM z-IETD-fmk) or caspase 9 (25 μM z-LEHD-fmk) (Enzo Life Sciences, Farmingdale, NY), followed by treatment with proteasome inhibitors as described.

To generate LN18 cells with stable knockdown of caspases, GIPZ lentiviral shRNA was obtained from GE Healthcare (Buckinghamshire, England) with sequences targeting caspase 2 (CAGACATCTCCTTGCACCG), caspase 8 (TTCTTAGTGTGAAAGTAGG), and caspase 9 (TGTCGTCAATCTGGAAGCT). Lentiviral infection of LN18 cells was performed using a Trans-Lentiviral Packaging Kit from Thermo Fisher Scientific (Waltham, MA) followed by puromycin selection.

### Glutathione measurement

Cells were washed 1× in PBS and resuspended in 1 mL PBS. Next, 2 μL monochlorobimane solution (2.2 mg monochlorobimane in 194.12 μL acetonitrile) was added to each sample. Samples were vortexed and incubated at 37 °C for 15 min. The reaction was halted by adding 50 μL trichloroacetic acid and vortexing. Samples were spun for 5 min at 10,000 rpm, and 1 mL supernatant was added to a glass tube containing 1 mL dichloromethane. Glass tubes were vortexed and centrifuged for 2 min at 3,500 rpm. For each sample, 200 μL of the top aqueous layer was plated in duplicate wells in a white 96-well plate. Fluorescence was read on a Gemini EM Microplate Reader (Molecular Devices, Sunnyvale, CA, USA) at an excitation of 360 nM and an emission of 460 nM. Glutathione concentrations were determined by comparing samples to a standard curve composed of varying concentrations of glutathione ethyl ester dissolved in PBS.

### Intracranial mouse xenograft model

The Institutional Animal Care and Use Committee at the University of Texas MD Anderson Cancer Center approved all experimental procedures (Protocol # 030402934) in accordance with the Animal Welfare Act and per recommendations of the Association for Assessment and Accreditation of Laboratory Animal Care. An intracranial guidescrew model of GBM was used, as previously described^43^. We implanted 500,000 U87 GBM cells though a guidescrew in 5-week-old female athymic nude mice (Experimental Radiation Oncology, MD Anderson, Houston, TX) and let tumors develop for 1–2 weeks (as noted in Results). Mice were then injected intraperitoneally with 1 mg/kg BTZ, 0.15 mg/kg MRZ, and/or 50 mg/kg vorinostat in dosing schedules outlined in the Results. For lysates, the tumor sections were frozen in liquid nitrogen, then homogenized by vortexing with zirconia/silica beads (Biospec, Bartlesville, OK, USA) in lysis buffer (20 mM Tris, pH 7.5, 0.1 mM EDTA, 20% glycerol, and 0.05% NP-40). For IHC analysis, samples were preserved in formalin, then paraffin-embedded.

### Immunohistochemistry

Proteins were detected in tissues using antibodies for p21 (sc-6246, 1:50 dilution: Santa Cruz, Dallas, TX) or cleaved lamin A (2035, 1:100 dilution: Cell Signaling, Beverly, MA). After incubation with secondary antibodies (for p21: biotinylated rabbit-anti mouse for 15 min, [Accurate Chem, Westbury, NY]; for lamin A: anti-rabbit horseradish peroxidase for 30 min [Dako, Glostrup, Denmark]), slides were incubated with Tablet DAB (for p21, Sigma) or Dako DAB (for cleaved lamin A). Slides were counterstained with hematoxylin. The number of positively stained cells was counted and averaged for 5 fields (40×).

### Statistical analysis

Values are given as the mean ± standard error of the mean, with all experiments performed at least in triplicate. Comparisons were made using Student’s *t-*tests performed using GraphPad Prism software, version 6 (GraphPad software, La Jolla, CA). P-values < 0.05 were considered significant. Synergy was determined based on DNA fragmentation assays using CalcuSyn software (Biosoft, Cambridge, United Kingdom), with combination index (CI) values <1 considered synergistic.

## Additional Information

**How to cite this article**: Manton, C. A. *et al.* Induction of cell death by the novel proteasome inhibitor marizomib in glioblastoma in vitro and in vivo. *Sci. Rep.* **6**, 18953; doi: 10.1038/srep18953 (2016).

## Supplementary Material

Supplementary Data
