# Supplementary material for: Induction of cell death by the novel proteasome inhibitor marizomib in glioblastoma in vitro and in vivo
Source: Sci Rep. 2016 Jan 25;6:18953. doi: 10.1038/srep18953 (PMC4726202; doi:10.1038/srep18953)
Supplement: Supplementary Data [file srep18953-s1.pdf]

Induction of cell death by the novel proteasome inhibitor marizomib in glioblastoma *in vitro* and *in vivo*

Running Title: Death induction by marizomib in GBM

Christa A. Manton, Ph.D., Blake Johnson, Ph.D., Melissa Singh, Ph.D., Cavan Bailey, M.S., Lisa Bouchier-Hayes, Ph.D., and Joya Chandra, Ph.D.*

Supplementary Information

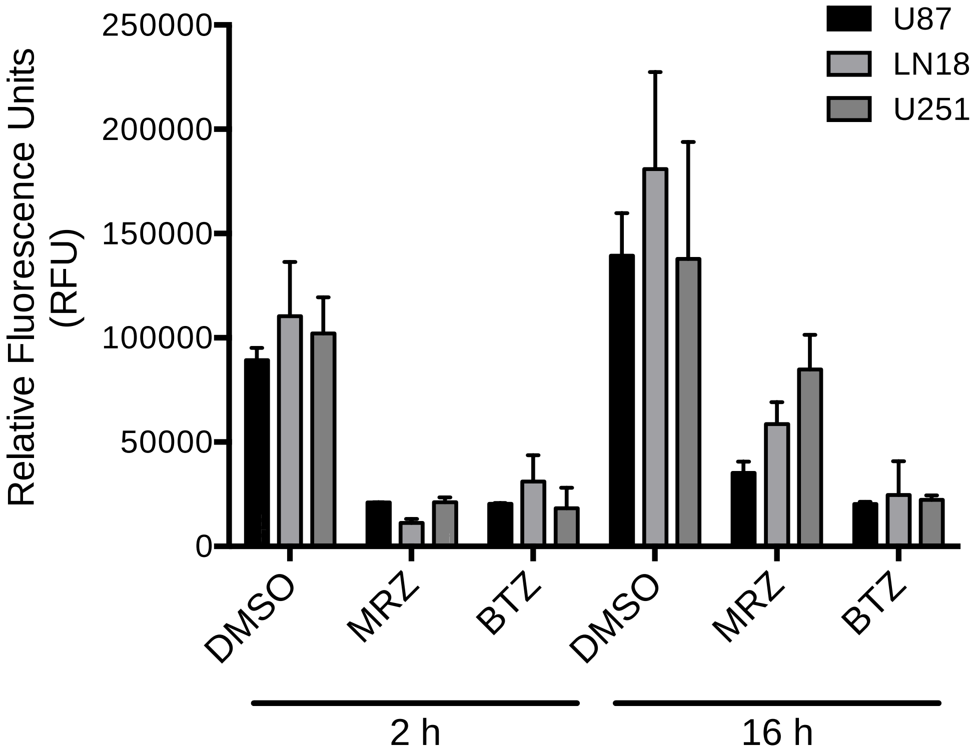

Supplemental Figure 1: Unstandardized chymotrypsin-like activity data shows that DMSO treatment does not significantly affect proteasome activity. GBM cell lines were treated for 2 or 16 h with 25 nM MRZ or BTZ, followed by analysis for chymotrypsin-like proteasome activity. This is the unstandardized version of data shown in Figure 2A.

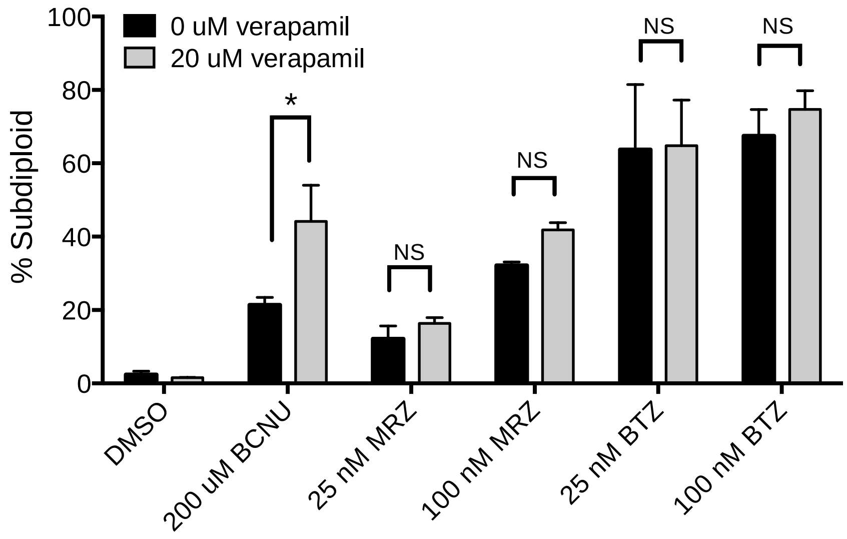

Supplemental Figure 2: Drug efflux through P-glycoprotein does not affect proteasome inhibitor efficacy. LN18 cells were pre-treated for 30 min with verapamil, followed by treatment with proteasome inhibitors or the known P-glycoprotein substrate BCNU for 48 h. Cells were stained with propidium iodide, and DNA fragmentation was assessed. Error bars represent the standard error of the mean for N=3 experiments. NS=not significant, *p<0.05.

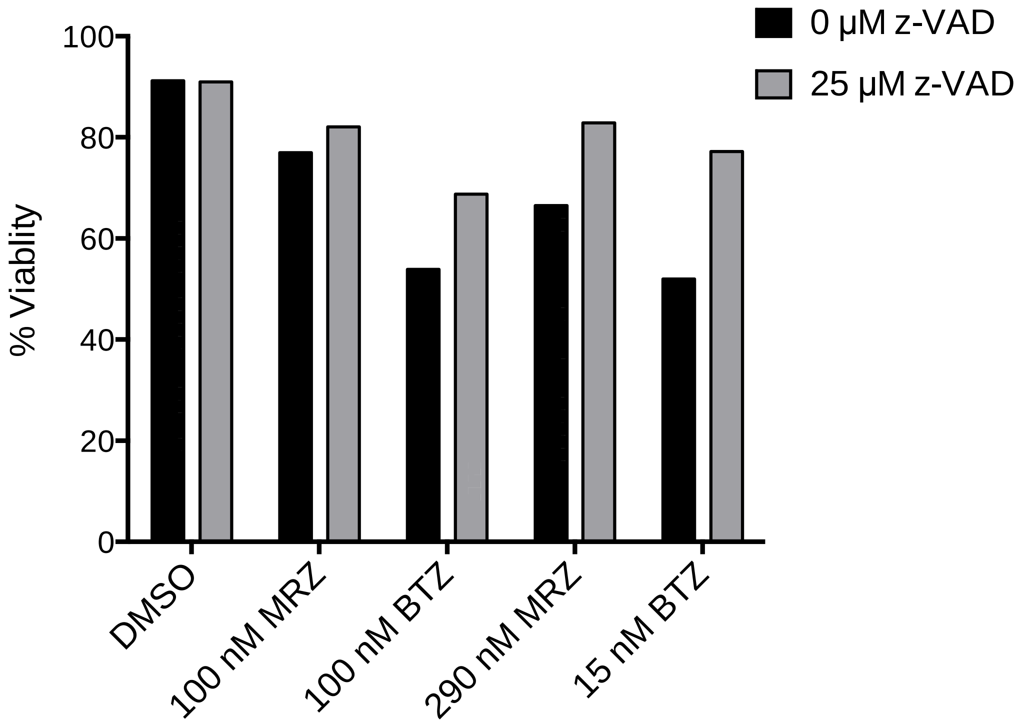

Supplemental Figure 3: Caspase inhibition increases viability following proteasome inhibitor treatment. LN18 cells were pre-treated 30 min with the pan-caspase inhibitor z-VAD, followed by 48 h treatment with proteasome inhibitors. Viability was measured by trypan blue exclusion.

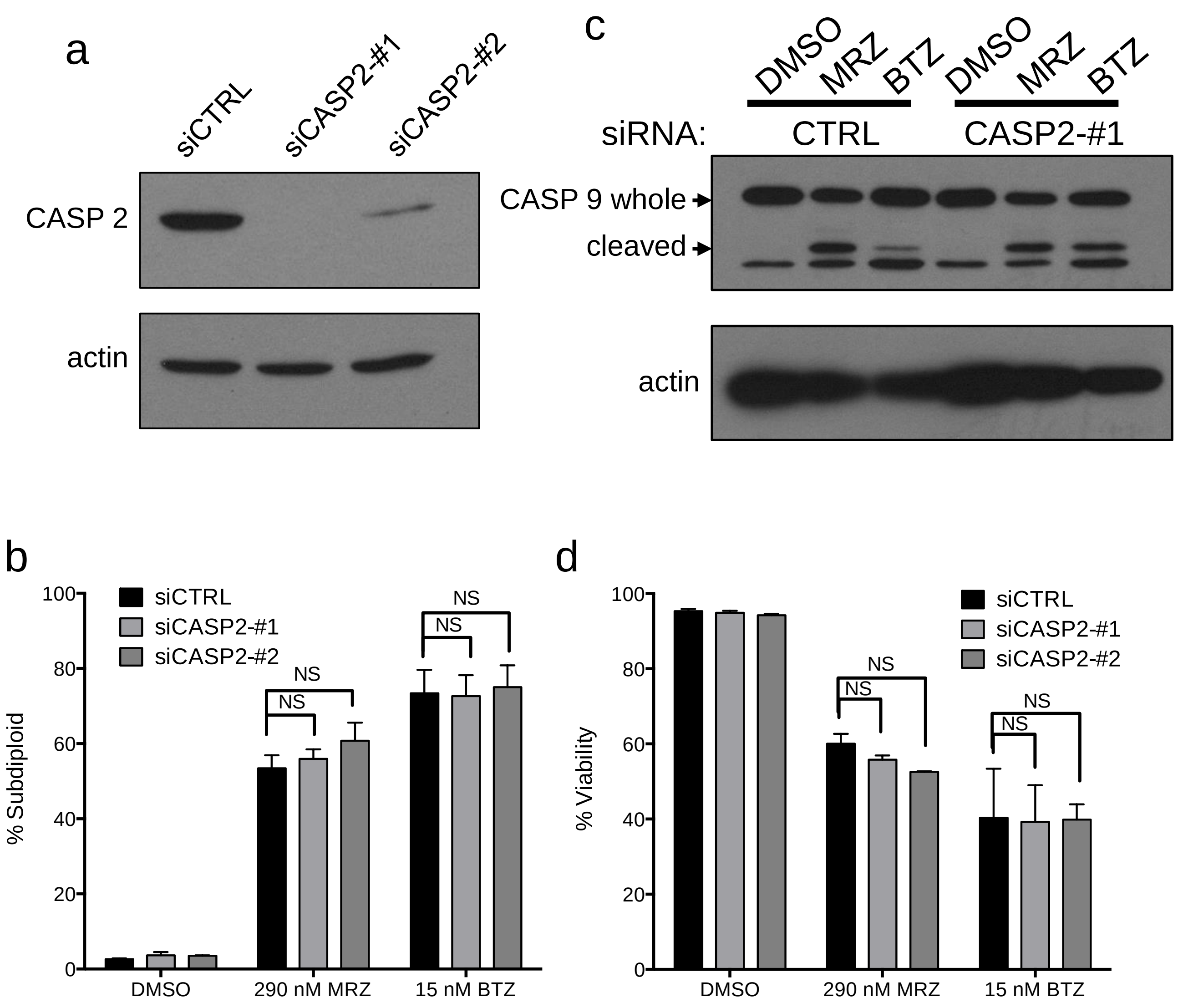

Supplemental Figure 4: Knockdown of caspase 2 with siRNA does not effect sensitivity to proteasome inhibitors. A) Western blot for caspase 2 after lipofection of LN18 cells with siRNA for caspase 2. B&D) Twenty-four hours following transfection with siCASP2, LN18 cells were treated for 48 h with proteasome inhibitors. Cells were analyzed for DNA fragmentation by propidium iodide staining (B) or viability by trypan blue exlusion (D) (NS = not significant). C) LN18 cells transfected with siCASP2 were treated 16 h with 290 nM MRZ or 15 nM BTZ, and caspase 9 cleavage was assessed by Western blot.
